# Overreaction to non noxious vascular stimuli in migraine and effect of NMDA receptor blockade

**DOI:** 10.1186/1129-2377-14-S1-P111

**Published:** 2013-02-21

**Authors:** M Nicolodi

**Affiliations:** 1 Foundation Prevention and Therapy Primary Pain, Italy

## 

In ‘94 we enlighten vascular hyperalgesia/allodynia in migraine (M) sufferers [1]. Central pain is defined as “spontaneous pain and painful overreaction to stimulation”. Delayed painful sensation was also described in so called hyperpathia following partial lesions in the CNS. The aim was to stress possible overreaction and its NMDA antagonists blockade after non noxious stimulation of vein walls induced as elsewhere described. Procedure: a sharp stretch of vein walls, innocuous in controls, was induced in M sufferers. Delayed overreaction might be reported and scored on a 0-10 VAS in 189 M sufferers (101 females , 88 males, mean age 32.5 +3.8 SD) reporting 6-10 attacks A3 /month. It was planned to administer ketamine, specific non competitive antagonist at NMDA receptor sites, to M sufferers to observe possible overreaction. Tested M sufferers reported delayed overreaction ranging from 3 to 8 on a 0-1 0 VAS (mean 4.5 + 1.5 SD). Delayed overreaction lasted from 5 min to 4320 min, mean 210.22 min + 623 SD . The majority of sufferers reported overreaction till to 25 min after application of the stimulus revealing their visceral/vascular hyperalgesia/allodynia, 5 reported overreaction lasting 4320 min and 6 were ailed by overreaction for 5 mins. The duration of overreaction was directely related to the severity/ frequency of headache attacks. ANOVA failed in evidencing sex or M duration relationship, whereas a modest significativity (p>0.02) was related to high scores in Wang and Zung tests (cut off = 40). Ten days later the same stimulus was applied after 0,1 mg/Kg/ i.m. administration of ketamine, after a 3 days wash-out period. Patients reported neither allodynia/hyperalgesia or overreaction. The experience indicated the occurrence of overreaction in M. The partial deafferentation condition was inhibited by using a sub-anesthetic dose of ketamine. Thus, the drug likely acted by altering process of neural deafferentation-like related discharge.

## References

[B1] Nicolodi M Sicuteri R Coppola G Greco E Sicuteri F Visceral pain threshold is deeply lowered far from the head in migraine Headache 1994 14 12 19 10.1111/j.1526-4610.1994.hed3401012.x 8132435

